# Protecting small and sick newborn care in the COVID-19 pandemic: multi-stakeholder qualitative data from four African countries with NEST360

**DOI:** 10.1186/s12887-023-04358-7

**Published:** 2023-11-16

**Authors:** Rosie Steege, Hannah Mwaniki, Ifeanyichukwu Anthony Ogueji, Jitihada Baraka, Sangwani Salimu, Meghan Bruce Kumar, Kondwani Kawaza, Opeyemi Odedere, Donat Shamba, Helen Bokea, Msandeni Chiume, Steve Adudans, Chinyere Ezeaka, Catherine Paul, Laurent Banyira, Gaily Lungu, Nahya Salim, Evelyn Zimba, Samuel Ngwala, Alice Tarus, Christine Bohne, David Gathara, Joy E. Lawn

**Affiliations:** 1 Maternal, Adolescent, Reproductive & Child Health (MARCH) Centre, London School of Hygiene & Tropical Medicine, London, UK; 2 Liverpool School of Tropical Medicine, Liverpool, UK; 3 Aga Khan University, Nairobi, Kenya; 4 NEST360/APIN Public Health Initiative, Connal Road, Yaba, Lagos State Nigeria; 5 Department of Health Systems, Impact Evaluation and Policy, Ifakara Health Institute, Dar es Salaam, Tanzania; 6 Kamuzu University of Health Sciences, Blantyre, Malawi; 7 Malawi-Liverpool-Wellcome Trust Clinical Research Programme, Blantyre, Malawi; 8 Kenya Medical Research Institute, Wellcome Trust Research Program, Nairobi, Kenya; 9 Department of Pediatrics, Kamuzu Central Hospital, Lilongwe, Malawi; 10 Rice360 Institute for Global Health Technologies, Houston, Texas USA; 11 Kamuzu Central Hospital, Lilongwe, Malawi; 12 Academy for Novel Channels in Health and Operations Research (ACANOVA Africa), Nairobi, Kenya; 13 College of Medicine, University of Lagos, Lagos State, Nigeria; 14 Malawi Adventist University, Ntcheu, Malawi; 15 Department of Paediatrics and Child Health, Muhimbili University of Health and Allied Sciences, Dar es Salaam, Tanzania; 16 Ifakara Health Institute, Dar es Salaam, Tanzania

**Keywords:** Small and sick newborn care, Neonatal care, Health systems, COVID-19, Health systems shocks

## Abstract

**Background:**

Health system shocks are increasing. The COVID-19 pandemic resulted in global disruptions to health systems, including maternal and newborn healthcare seeking and provision. Yet evidence on mitigation strategies to protect newborn service delivery is limited. We sought to understand what mitigation strategies were employed to protect small and sick newborn care (SSNC) across 65 facilities Kenya, Malawi, Nigeria and Tanzania, implementing with the NEST360 Alliance, and if any could be maintained post-pandemic.

**Methods:**

We used qualitative methods (in-depth interviews *n*=132, focus group discussions *n*=15) with purposively sampled neonatal health systems actors in Kenya, Malawi, Nigeria and Tanzania. Data were collected from September 2021 - August 2022. Topic guides were co-developed with key stakeholders and used to gain a detailed understanding of approaches to protect SSNC during the COVID-19 pandemic. Questions explored policy development, collaboration and investments, organisation of care, human resources, and technology and device innovations. Interviews were conducted by experienced qualitative researchers and data were collected until saturation was reached. Interviews were digitally recorded and transcribed verbatim. A common coding framework was developed, and data were coded via NVivo and analysed using a thematic framework approach.

**Findings:**

We identified two pathways via which SSNC was strengthened. The first pathway, COVID-19 specific responses with secondary benefit to SSNC included: rapid policy development and adaptation, new and collaborative funding partnerships, improved oxygen systems, strengthened infection prevention and control practices. The second pathway, health system mitigation strategies during the pandemic, included: enhanced information systems, human resource adaptations, service delivery innovations, e.g., telemedicine, community engagement and more emphasis on planned preventive maintenance of devices. Chronic system weaknesses were also identified that limited the sustainability and institutionalisation of actions to protect SSNC.

**Conclusion:**

Innovations to protect SSNC in response to the COVID-19 pandemic should be maintained to support resilience and high-quality routine SSNC delivery. In particular, allocation of resources to sustain high quality and resilient care practices and address remaining gaps for SSNC is critical.

**Supplementary Information:**

The online version contains supplementary material available at 10.1186/s12887-023-04358-7.

## Background

Some 30 million newborns need hospital care each year, many of whom are preterm and extremely vulnerable [1]. Momentum to improve small and sick newborn care (SSNC) is building; the Sustainable Development Goals (SDGs) include a specific neonatal mortality target, with all countries aiming to reduce neonatal mortality to at least as low as 12 deaths per 1,000 live births [2] and the 2025 Every Newborn Action Plan (ENAP) coverage four target aims for ‘*80% of districts (or equivalent subnational unit) have at least one level-2 inpatient unit to care for small and sick newborns...*’ [3]. However, progress towards these targets has been slower than efforts to reduce maternal and child mortality [1]. The United Nations Inter-agency Group for Child Mortality Estimation indicates that neonatal mortality in 2021 for sub-Saharan Africa was 27 per 1000 live births with an absolute reduction of about 2 deaths per 1000 lives in the period 2015 -2021 [4]. Kenya, Malawi, Nigeria and Tanzania were estimated at 18, 19, 20 and 24 deaths per 1000 live births respectively in 2021 [4]. To date, SSNC in low- and middle-income countries (LMIC) has been undermined by health system weaknesses [5] and a limited focus on community participation and family-centred care. Efforts to work towards these targets must, therefore, include broader health system strengthening strategies to support health system resilience in the face of increasing health systems shocks.

Throughout 2020-2021, the COVID-19 pandemic led to large scale interruptions to health systems globally through reduced healthcare seeking and disruptions to health services provision [6–9]. Shocks – such as epidemics and forced displacement among others– are predicted to increase in both frequency and severity and will test health systems. The negative early effects of the pandemic on SSNC and maternity care have been well documented [6–13]. Country-specific quantitative evidence from Nepal and Uganda describes reduction in admissions with increased stillbirth rates and neonatal mortality rates during lockdown [9, 12]. Large-scale global surveys conducted as the pandemic unfolded in 2020 also highlighted widespread interruptions to family-centred care and disruptions in newborn care practices [6, 11, 13]. These included: closure of neonatal wards, reallocation of staff from newborn care to COVID-19 duties, early discharge of patients and compromised oxygen supplies. Evidence-based interventions such as kangaroo mother care (KMC) were reported to be postponed until COVID-19 status was known, discontinued or discouraged [6], despite the known benefit on mortality outcomes [8]. The pandemic also impacted on frontline health workers’ ability to provide high quality, respectful maternity care, with reportedly less family involvement, reduced emotional and physical support for women and increased exposure to medically unjustified caesarean section, among others [11]. These disruptions, even if only for a short time, could lead to additional deaths [14]. Modelled estimates of 118 countries using the Lives Saved Tool suggested that reductions in coverage (i.e. provision and utilisation of health services) of 15% for six months could result in 253,500 additional child deaths [14].

Adaptations to health service delivery were widely adopted to work within new guidance and manage the effects of the pandemic. Survey evidence from healthcare workers in LMIC on SSNC reported on potential enablers to support SSNC as: communication with communities; use of phones to counsel on KMC and breastfeeding after discharge; separate isolation wards; education and emotional support strategies for healthcare workers among others [6]. Similar mitigation strategies to maintain services for maternal, newborn, child and adolescent health were also identified in a global WHO report that covered March-December 2020 [15]. Despite this, there are limited qualitative studies exploring mitigation strategies for SSNC [16], and therefore limited contextual depth and detail from which to understand systems level strategies and adaptations.

NEST360 is an international alliance united to end preventable newborn deaths in 65 African hospitals in four African settings: Kenya (13 facilities), Malawi (38 facilities), Nigeria (7 facilities) and Tanzania (7 facilities). NEST360, together with governments, works to reduce neonatal deaths and strengthen health systems via four approaches 1) introduction of existing technologies designed for LMIC contexts, 2) training and upskilling, 3) efforts to improve information systems, and 4) quality improvement and mentorship approaches [17]. NEST360 conducted an interrupted time series analysis of 65 facility newborn units across four African countries [18]. Positively, this analysis showed no statistical evidence of mortality change at two interruption timepoints – March 2020 and April 2021 during the COVID-19 pandemic. This suggests mitigation strategies employed may have had a protective effect. NEST360 provided a platform to explore in detail the practices employed to protect SSNC during the pandemic, and the limitations of these.

### Aim and objective

This paper is part of a series of papers to inform scale up of high quality SSNC in LMIC.

Our objective was to illuminate actions taken to protect SSNC during COVID-19 across four different country contexts. This aimed to inform systems level mitigation strategies to improve SSNC resilience against health system shocks.

## Methods

### Study context

**Fig. 1:**
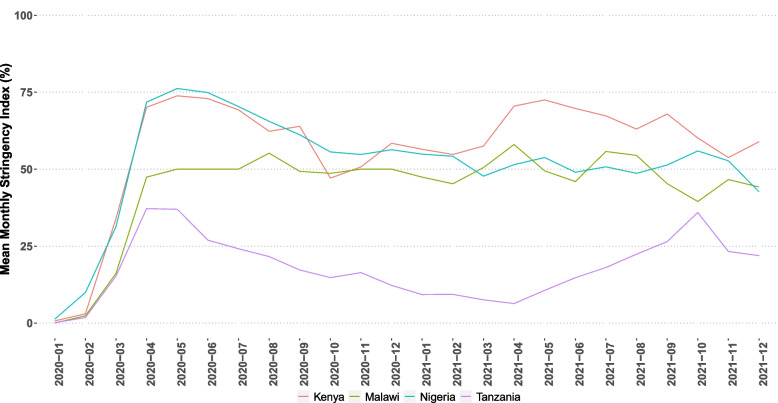
The Oxford Stringency Index summarised by month across four countries implementing with NEST360 (Malla *et al.* forthcoming)

COVID-19 was present from March 2020 in Kenya, Malawi, Nigeria and Tanzania (Additional file 1). Each context had differing national COVID-19 response measures that varied in severity. The Oxford COVID-19 Government Response Tracker (OxCGRT) measures the stringency level of government imposed COVID-19 protection measures that would have impacted healthcare seeking and delivery across different contexts [19]. The OxCGRT is computed by coding 23 variables which relate to containment and closure policies, economic policies, health system policies and vaccination policies etc. to produce a stringency score as a weighted average percentage [20]. Malla et al.’s (forthcoming) [18] analysis of the OxCGRT across the four countries from January 2020 to December 2021 demonstrates that policies seemed to be more stringent in Kenya and Nigeria at the start and less so in Malawi and least stringent in Tanzania. Whilst Nigeria’s stringency index fell from the outset of the pandemic to a level aligned with Malawi; Kenya’s remained comparatively high. Tanzania’s remained the least stringent throughout. Active implementation of policies appeared to have commenced in March and started becoming more stringent around April 2020 (Fig. 1).

### Study design

We conducted qualitative research across the four study countries implementing with NEST360 to explore multi-level mitigation strategies adopted during the COVID-19 pandemic.

**Fig. 2:**
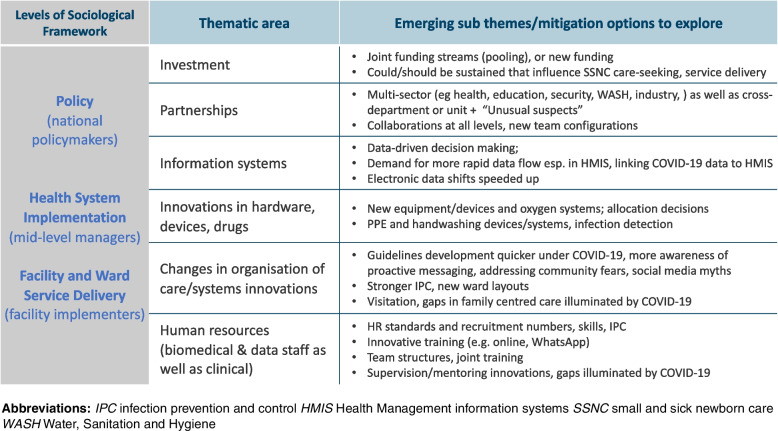
Framework of emerging themes to inform tool development – adapted from (Rao *et al.* 2021)

A conceptual framework published by Rao *et al.* 2021 that highlighted emerging themes of mitigation strategies across a socio-ecological framework was adapted (Fig. 2) to guide the design of data collection tools [6]. Standardised topic guides for in depth interviews (IDIs) and focus group discussions (FGDs) were co-developed based on themes from the framework [6] with NEST360 partners (including SSNC clinicians, policymakers and biomedical technicians) and government officials across the four countries. Government officials and NEST360 partners inputting to the guides were not included in the research. Guides provided a common frame across the four country teams (supplementary file). Questions explored themes of policy development, funding and partnership, changes in organisation of care, human resources, innovations in hardware and information systems. In Tanzania topic guides were translated into Swahili for interviewing and in Malawi into Chichewa.

### Sampling strategy and recruitment

The study population pertained to those working in the field of SSNC – including facility staff (e.g. hospital directors, neonatologists, paediatricians, neonatal nurses, biomedical engineers, information systems staff) at public facilities with NEST360, as well as external funders, device distributors and key stakeholders working at international and national policy levels (e.g. WHO, UNICEF). A sampling framework was collaboratively developed by core NEST360 staff to identify respondents representing multidisciplinary health systems actors at all levels. See Table 1. Purposive sampling was used to capture a range in cadres, years of experience, geographic location, gender and heterogeneity of facilities.

**Table 1:** Summary In Depth Interviews (IDI) and Focus Group Discussion (FGD) respondents by country and cadre

**Respondent**	**Kenya**		**Malawi**		**Nigeria**		**Tanzania**	
**Number of facilities sampled**	**4**		**5**		**7**		**5**	
**Interview type**	**IDIs**	**FGDs**	**IDIs**	**FGDs**	**IDIs**	**FGDs**	**IDIs**	**FGDs**
National policymakers	5	-	2	-	4	-	2	-
International stakeholders working in SSNC (e.g., WHO, UNICEF, USAID, Save the Children)	1	-	6	-	5	-	2	-
NEST360 team (includes M&E manager, data officers, biomedical engineers)	4	-	-	1 (*n*=5)	2	1 (*n*=4)	5	-
Funders - national	1	-	2	-	-	-	3	-
Device distributors	1	-	1	-	2	-	1	-
Mid-level management	-	-	-	1 (*n*=5)	3	-	3	-
Hospital administrator	4	-	5	-	-	-	2	-
**Facility Staff working in SSNC:**						**Mixed cadres 2(n=12)**		
Biomedical engineer	5	1(*n*=6)	5	1(*n*=5)	2	-	4	1 (*n*=8)
Clinician (Drs/Nurses)	13	1(*n*=6)	10	2(*n*=6)	2	2(n=5-7)	7	1(*n*=14)
HMIS officers	3	-	5	1(*n*=5)	-	-	3	-
Medical officer in charge	3	-	-	-	-	-	4	-
**Sub-total**	**40**	**2**	**36**	**6**	**20**	**5**	**36**	**2**
**Total IDIs ** ***N*** ** = 132 Total FGDs N=15**								

**Abbreviations:** *IDI* In Depth Interview, *FGD* Focus Group Discussion, *HMIS* Health Management Information System, *M&E* Monitoring and Evaluation, *SSNC* Small and Sick Newborn Care

In all countries, we secured letters of support from relevant authorities and institutions. We introduced study objectives to facility personnel who helped identify participants. In some cases, snowball sampling was also employed to identify further participants of interest.

### Data collection

Data collection took place from September 2021 - November 2021 (February 2022 - August 2022 in Tanzania). We undertook IDIs *n*=132, FGDs *n*=15 (see Table 1 for breakdown). Interviews were conducted by experienced local qualitative researchers without prior involvement in NEST360 (IAO, HM, SS, LB, GL, JB) who were trained prior to data collection on the aims, objectives, ethics and interviewing skills (RS). Weekly country-specific reflexivity and de-brief sessions were held. In addition, weekly multi-country debriefs were held to compare and contrast across contexts, clarify concerns and ethical dilemmas, triangulate across data, identify saturation of themes and further lines of inquiry. Data were collected until saturation was reached. Interviews were digitally recorded and transcribed verbatim to support analysis.

### Data analysis

We read and familiarised ourselves with the transcripts to collaboratively develop a common coding framework. The coding framework included both a priori themes from the topic guide, as well as inductive themes emerging from the data. We discussed and refined themes and referred to our reflexivity notes to support analysis. We used NVivo software (version 1.6.1) to code the data. A meeting in Nairobi (December 2021) allowed the opportunity to refine the coding framework collectively and present the adapted framework with the wider NEST360 team as an internal validation exercise. Although data in Tanzania were collected at a later stage, lead researcher in Tanzania (JB) was involved in co-development of the coding framework and was able to reflect on and contribute observations from Tanzania. Once the data from Tanzania were collected, the coding framework was revisited although no changes were required. Any variances in coding were discussed and resolved with RS, who checked a sample of all coding to ensure consistency. Facility-level discussions were also held as part of an external validation exercise and contributed to participant checking.

### Ethics

Ethical approval was granted by London School of Hygiene and Tropical Medicine - 21892. Study site ethical approval was granted by The Maseno University Ethics Review Committee, Kenya MSU/DRPI/MUERC/00810/1, National Commission for Science, Technology and Innovation, Kenya NACOSTI/P/22/15751, National Health Science Research Committee, Malawi 2463, Lagos University Teaching Hospital, Nigeria ADM/DCST/HREC/APP/3487, University College Hospital Ibadan, Nigeria UI/EC/20/0713, National Health Research Ethics Committee Nigeria NHREC/01/01/2007, Ifakara Health Institute, Tanzania IHI/IRB/01-2021, Muhimbili University of Health and Allied Sciences and National Institute for Medical Research, Tanzania MUHAS-REC-12-2019-072, National Institute for Medical Research, Tanzania NIMR 3405.

All participants gave written and/or oral informed consent to participate

## Results

**Fig. 3:**
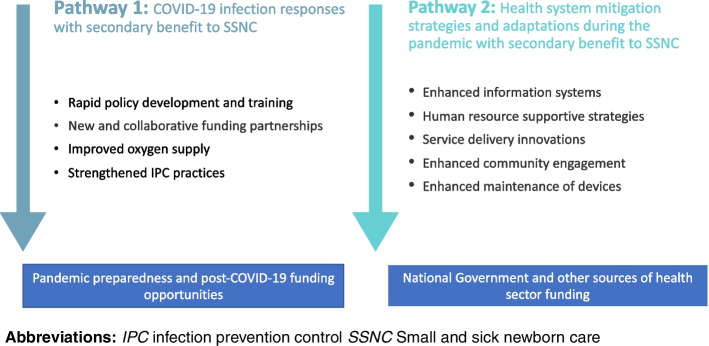
Two pathways of pandemic response that protected SSNC

The qualitative data reveals there were two pathways to manage the ongoing COVID-19 pandemic that impacted on SSNC (Fig. 3). The first pathway relates to direct responses to COVID-19 infection, with a secondary benefit for SSNC. The second pathway demonstrates general health systems adaptations taken within facilities during COVID-19, which may also have a secondary benefit to SSNC services. Further, chronic health systems weaknesses impacting delivery of SSNC and areas to improve sustainability were highlighted by our respondents. These are discussed in detail below and summarised in Table 2.

**Table 2:** Key mitigation strategies employed during COVID-19 and remaining gaps

Levels of Socio-ecological framework	Thematic area	Key mitigation strategies employed	Key gaps to address
POLICY (national policymakers)	Rapid policy development	• Rapid guidelines development and implementation • Responsive feedback mechanisms and flexibility • Continual updating of policies/guidelines • Training on new policies conducted	• Top-down policy development process lack insight of healthcare workers and communities • Lack of specific guidance for neonates • Minimal funds to support training (Tanzania)
	New collaborations and investment	• Pooled funding streams and new domestic funders • Government funds available for PPE • Local philanthropic funding calls/ community based assistance • Multi-sector collaborations (Education, health, WASH)	• Lack of specific funding allocations for SSNC • Limited cross departmental collaboration • Need to strengthen community engagement and participation
HEALTH SYSTEM IMPLEMENTATION(mid-level managers)*	Information systems	• Enhanced demand for data • Electronic data shifts speeded up • Increased ownership and accountability of data at facility level	• Culture of data use missing • Lack of ownership of data at facility level • Insufficient equipment to support shift to electronic data (e.g. hardware) • Data not used in funding proposals
	Devices	• Hastened roll out of O2 due to pandemic • New equipment/devices and oxygen systems • Shared O2 allocation decisions within and between hospitals • Good supply of PPE and handwashing devices/systems • Use of telemedicine for training of equipment • Pre-emptive planning for supplies (O2 etc) • Emphasis on planned preventive maintenance	• Lack of toolbox for equipment • Lack of locally available spare parts • Slow procurement process • Equipment shortages • Lack of training/ proper manuals • Minimal coordination between biomeds and clinicians • Lack of airtime for biomeds to support telemedicine
FACILITY AND WARD SERVICE DELIVERY(facility implementers)*	Service delivery	• Stronger IPC focus • New ward layouts (inborn/outborn, by dependency, by COVID status) • New newborn wards created • Visitation times and numbers limited • Shift to telemedicine for follow up • Changes in opening hours	• Limited space in wards • Early discharge to community • Staff rotations
	Human resources for health (HRH)	• Training on COVID-19 and IPC • Innovative training & troubleshooting (e.g., online, Whatsapp) • Financial support during COVID-19 • Use of locum staff/ students • Supervision/mentoring • Counselling services • Transport and insurance provided (Kenya)	• HRH shortages • Poor mentorship • HRH wellbeing support lacking • No clinician supervision structures • Few specialists • Need to train more biomeds • Shifts by experience (biomeds) • No vaccination offered to health staff (Malawi)
COMMUNITY LEVEL	Community engagement	• Downward referrals for non-complicated deliveries • Proactive messaging to communities • Working with CHWs, community leaders to support messaging for SSNC and IPC	• Need to strengthen referral systems • Need to strengthen primary healthcare units – including equipment

**Abbreviations:** *IPC* Infection prevention and control, *PPE* Personal protective equipment, *SSNC* Small and sick newborns, *CHWs* Community health workers, *O2* Oxygen

### Pathway 1: COVID-19 specific responses with secondary benefits to SSNC

#### Rapid policy development and adaptation

Respondents across the four countries reported that COVID-19 specific policy changes were rapidly developed and implemented in direct response to the pandemic – this occurred at the global and national levels, as well as within facilities. These policies were flexible and evolved as “*the pandemic unfolded*,” (IDI, Policy maker, Man, Malawi). It was noted in Tanzania that there was opportunity to adapt and ‘individualise’ guidance at the tertiary hospital (zonal) level. Implementation was also reported to be efficient via downwards cascading of information and trainings. These adaptations may have had indirect benefits to SSNC (via improved IPC for example).

‘I would say we responded promptly and we responded efficiently… we watched the trend that the pandemic was taking and the protocols given from WHO, cascaded down to National Centre for Disease Control, and given to us by the Lagos State government which we promptly instituted in our own facility... So, we senior facility staff had to ensure that everyone complied with the protocols, we also educated everyone to make sure that as senior personnel, the necessary information that the junior personnel needed are available...’ (FGD - Paediatrician, Woman, Nigeria)

A critical gap across all countries, however, was the lack of specific COVID-19 neonatal guidance and a robust public health emergency plan for neonates. This also meant staff and oxygen were pulled away from neonatal units at the outset of the pandemic. This may have been a consequence of the normative governance model that limited insights of the frontline actors and their interactions - respondents in all countries noted there were gaps in hospital staff involvement in guideline development, which was often top-down in design and rarely consulted various levels of facility staff. Staff would end up looking to paediatric COVID-19 health guidelines in lieu of neonatal guidelines, but the flexibility to evolve the guidance rapidly helped mitigate this oversight.

‘...Even for older children (policy) was not that detailed, you find it probably just a paragraph, if you can see those green booklets over there (pointing) those were the national guidelines for COVID but the section for paediatric is not that extensive and just covers paediatric in general not neonatal’. (IDI, Hospital Director, Tanzania)

‘R: management at this hospital will sat down and formulated some of the guidelines, but I think, you know, this is a new thing so most of the things were over looked and maybe we EVEN didn’t consider the neonate itself…

I: Alright, so actually realising that some of the issues were missed out, did you do anything to address to that?

R: Yeah!… We are improvising, for example in isolation ward we identified a certain room where we took like a heater put in that, a phototherapy machine put in that, but also a CPAP… as an improvised room there is a window there, but you know in nursery we are not supposed to have windows.’ (IDI, Neonatal Nursing Officer, Woman, Malawi)

### New and collaborative funding partnerships

It was narrated that the funding environment changed during COVID-19. Governments released funds specifically for the purposes of providing PPE to facilities and there were reports of philanthropic donations from communities that extended beyond the ‘usual’ donors. For example, in Kenya and Nigeria, national banks and non-governmental organisations supported funding for oxygen systems and donated PPE. In Nigeria and Malawi, private philanthropists and community-based organisations were mentioned as supporting financially and community-based organisations were mentioned in Tanzania. In-kind donations were also highlighted. These were also decentralised directly to facilities, which was reported to be a positive across Malawi, Kenya and Nigeria, with a particular benefit for primary or secondary facilities. Regrettably, these donations were mostly one-time or short-term donations when COVID-19 first was declared a pandemic in 2020 – giving governments and hospitals a false sense of investments that were not sustained. In Tanzania, all COVID-19 funding was initially provided by the government until a leadership change within government meant external sources of funding for COVID-19 activities were accepted. Prior to that, funds to implement timely training on COVID-19 guidelines and policies from the Ministry of Health had been a reported bottleneck. To mitigate this some of the hospitals leveraged internal funding for trainings and COVID-19 advocacy.

‘The paying of staff compensation, need for cars and fuel to facilitate dissemination of the guidelines to reach the districts you must have financial resources, therefore financial aspect was a dilemma.’ (IDI, Hospital Director, Tanzania)

Pooled funding streams and strong partnerships from international donors in the COVID-19 response was a reported benefit. Leading to increased multi-sectoral collaboration and harmonisation across governing bodies, facilities, and sectors in all contexts:

So, everyone plays a role, activities were coordinated, even the support for oxygen was also involved. However other actors were coming in to give an update on how far they have gone with oxygen support, the oxygen plants, the oxygen cylinder, who is supporting what and at which hospitals, where are the gaps and who can actually cover that gap….’ (IDI, Funder, Woman, Tanzania)

In Kenya, the supportive environment that COVID-19 created for funding proposals and swift action was highlighted. On the contrary, respondents in Malawi reported a lack of support for proposals in SSNC - limiting institutionalisation.

### Improved oxygen supply

Linked to funding availability, there was increased support for funding oxygen systems in all countries, which had a direct benefit for SSNC.

‘A lot of opportunities [for SSNC] came up. Due to the government seeing the lapses that COVID brought, supports are now being offered to health facilities in the area of oxygen security… there are now increases in the number of oxygen cylinders in facilities.” (FDG, biomedical engineer, Man, Nigeria)

‘…we have used resources of COVID to strengthening the health systems which directly benefits the maternal and newborn health activities, in terms of improving WASH services, WASH infrastructure, in terms of providing of oxygen for example the provision of oxygen now we need it for COVID right? But oxygen is a determinant in the survival of newborn... There has been lack of oxygen in many locations, we have seen many newborns dying, but now many locations have oxygen concentrators, oxygen cylinders and continued supply of oxygen so the resources have been moved from here and there.’ (IDI, Funder, Man, Malawi)

This contrasted with the early days of the pandemic in Malawi, where initially oxygen had been ‘borrowed’ from neonatal wards until new rules were put in place to stop this.

Some facilities that had previously relied upon oxygen cylinders suddenly found there was support for hastening the rollout of piped oxygen:

‘we really wanted to have piped oxygen and when COVID came in, it fast tracked that, now we are able to have piped oxygen in the ward, which was actually a major problem before.’ (IDI, Neonatal Nurse, Woman, Kenya)

In Tanzania, one respondent spoke of the government’s strategic investment in oxygen plants within seven regional hospitals to produce a consistent supply. These oxygen plants were able to produce enough oxygen to sell to neighbouring facilities and generate income for the hospitals. There was also specific rapid guidance developed on the use and maintenance of oxygen devices during the period.

‘Initially during the very first COVID wave, the government worked on oxygen service to be available. Oxygen plants were installed in seven regional referral hospitals and the government has continued to expand the investment to other hospitals. The staff were trained to ensure the services continues… government funding was set up to keep the service consistent at all times’ (IDI, biomedical engineer, Man, Tanzania)

‘we can fill almost 30 cylinders a day and be used for emergency and for sale’ (IDI, biomedical engineer, Woman, Tanzania)

### Strengthened IPC practices

Strengthening IPC in response to COVID-19 occurred across all countries. COVID-19 presented an opportunity to upgrade protocols on IPC, which had a subsequent benefit for neonatal care.

‘You know one of the ways of preventing COVID spread is adequate and appropriate hand hygiene, and hand hygiene has more to offer neonatal care than just wanting to prevent COVID.’ (IDI, Paediatrician, Woman, Nigeria)

‘… nowadays we really try washing completely like those incubators … so we have seen the other infections going down.’ (IDI, Neonatal Nurse, Woman, Kenya)

Strengthened IPC was reported to occur throughout facilities and in multiple ways, though a lack of space in neonatal wards was said to limit physical distancing measures. Examples of increased PPE use and sanitising/handwashing were emphasised and expected among health workers and families, but there were many more novel practices employed - such as restricting visitors to neonatal wards, installing one-way systems for people traffic within facilities, limiting the amount of people allowed in staff break rooms and adjusting ward layouts.

### Pathway 2: Health system mitigation strategies and adaptations during the pandemic with secondary benefits to SSNC

#### Enhanced information systems

The rapidly evolving pandemic demanded timely data and an increased use of data to support decision making. This reportedly improved data quality for SSNC. In Nigeria the environment encouraged a shift to electronic data collection and supported timely data collection during the pandemic. The success of this, however, was limited by a lack of hardware.

‘Before this COVID-19, we used to collate data within an interval of every three days, but now, we are collating data on daily basis because we won’t like a situation where we miss out of a vital information on any patient that is why we do this on daily basis now.’ (FGD, HMIS, Woman, Nigeria)

“The COVID pandemic required us to move to electronic means of data recording; however, we are incapacitated to adjust to this demand in electronic shift due to insufficient laptops for work, and this has limited our productivity at this time.” (IDI, HMIS, Man, Nigeria)

In Malawi it was noted that the reporting requirements did not change, but that the influx of funding allowed for additional analysis. In Kenya specifically, data ownership and use were reportedly lacking. The need to build understanding of the benefits of data capture across all facility staff was reported as a critical gap. The governance approaches in Tanzania may have led to increased accountability for data use and a culture of data ownership. COVID-19 data were not shared outside of the Ministry of Health but there was increased training on data collection and use. Further, the Ministry of Health were reported to be involved in spot checking the quality of the data collected.

‘Also gave an awareness that we as an institution or we as a data department got training on how to analyse data and create more accurately plans unlike in the beginning where we were seeing the data just as normal information but with this they added to us power that data are of important.’ (IDI, HMIS, Man, Tanzania)

### Human resource adaptations and supportive strategies

COVID-19 presented obvious challenges to human resources. Respondents across all contexts spoke of staff absences, relocations and burn out. In Nigeria and Kenya, this reportedly led to the shutting down of a facility or unit. Additionally, nurses working in SSNC were initially pulled out to work on COVID-19 isolation centres, which in Nigeria was linked to not having a robust public health emergency plan. To manage these challenges and protect SSNC, all facilities reported pulling together as a team and task-sharing. Staff collaborated across wards and roles in new ways. Some facilities supplemented staff shortages via locum staff, student staff or government supplied staff. There were examples of distance-based training, increased supervision and opportunities to attend virtual trainings and upskill in a way that was not possible prior to the pandemic.

‘COVID has had enormous impact when it comes to issues such as staffing… people working in a designated facilities are pulled out of those facilities to work in these isolation centres … [this] could negatively impact the staff because they are more frequently rostered… this could lead to burnout… this negatively impacts the care of small and sick newborn…On the other side…in terms of capacity building, COVID enabled access to a lot of online training and information that could help improve individual staff knowledge and skills which could make a better person and enable them to deliver care in more appropriate way..’ (IDI, Paediatrician, Woman, Nigeria)

‘Some staff had a relative… had a COVID (case) in the household so it created a gap. So what we did we beefed up staff but those new recruits… so had an intensive orientation plan for the first two weeks, where we would have a unit matron a qualified in the nursery and new recruit to orient them on the protocols, the machines and everything…So I think that orientation plan helped us.’ (IDI, Neonatal Unit Matron, Woman, Malawi)

Policies implemented to support health workforce morale, safety and wellbeing were also noted. All countries had some provision of financial support to health workers working through the pandemic. Though it was reported that in Tanzania not all health workers received these funds and in Nigeria and Kenya these payments were reported as delayed. One respondent in Tanzania highlighted their desire for funding to be ringfenced to incentivise frontline health workers in times of crises*.* In Malawi however, there were concerns from respondents at various levels of the health system of the sustainability of incentivising COVID-19 care:

'the one thing that.. worries me for the future in terms of sustainability post COVID-19 is that COVID-19 has been associated with money, so if you work with a COVID-19 patient you should be paid, health workers all want to receive something… but these are things that we as health workers are supposed to do whether there is money or not, so that is my concern.’ (IDI, Hospital Director, Man, Malawi)

One Kenyan facility also implemented supportive policies to provide staff and their families with insurance for COVID-19 testing and medication. This was in conjunction with the provision of transport to the hospital via common routes to minimise staff exposure and comply with travel restrictions. A psychosocial support system was also implemented to support health workers serving through the pandemic. Together these measures had a positive impact on morale and demonstrate the critical role of supportive leadership in empowering health workers during uncertain times.

‘…they brought psychologists to have sessions with patients and staff to help in the anxiety and fear so at least the fear was allayed and the confidence was boosted, you know nurses are the most who come into contact with the patients first most of the time, so they were now able not to run away from the patients but know the mode of transmission and prevention so they were able to give services with confidence.’ (IDI, Hospital Administrator, Woman, Kenya)

In all contexts, mentoring was flagged as a critical gap in human resource support to address staff burn out. Further, in Malawi, one stakeholder stressed the importance of granting staff access to vaccination (which was not done), to ensure staff felt safe and supported to provide care during the pandemic.

### Service delivery changes and innovations

Mitigation strategies were also employed to support continuity of care. Telemedicine was reported to be used to protect SSNC follow up in communities across all contexts and alleviate health worker fears. WhatsApp and Zoom were used to take patient calls and consultations, usually at healthworkers’ cost. Whilst this could confer benefits to routine newborn care by supporting early discharge and limiting the chance of hospital-acquired infections, it came with a warning from an older clinician in Kenya who highlighted it was not comparable to seeing a patient in person as different diseases can present similarly.

Yes, it is a good thing, it is an easy thing one can do maybe with the new generation but for those of us who were bred in the old analogue ways, I always believe in it is always good to touch a patient. (IDI, Paediatrician, Woman, Kenya)

Further notable limitations of telemedicine were: poor network coverage, lack of clinician and patient familiarity with software, and a lack of airtime provision to staff using telemedicine for their routine work. In some cases, the financial burden was put on senior staff in hospitals to provide airtime for staff members, who narrated the choice young doctors make in purchasing PPE to protect themselves, or airtime to protect their patients:

‘Well, yes, I will say we use zoom to see babies now. When the mothers call me to say ‘doctor, my baby’s breathing is sounding somehow’ I will say okay, can we do WhatsApp video call now, let me look at the baby. Sometimes, we tell the mothers that if they have complaints, take a video clip, send it to me electronically, via WhatsApp, we look at it, discuss it, and give feedback to the mother… However, poor internet service and high internet service cost have been additional challenges. I particularly feel bad for my younger doctors that have to use their money to purchase mobile data while also purchasing nose masks because I can’t buy it for them all as we don’t have provisions for such so it’s extra burden on them.’ (IDI, Paediatrician, Woman, Nigeria)

Ward layouts were also reorganised to protect newborns. Some examples employed by various facilities were separating inborn and outborn babies, separating by COVID-19 status, or by dependency. In Kenya, changes with regards to dependency was also reported to support staff morale who could mentally prepare for a day working on a high dependency ward.

‘... So, if you are deployed in room 6, you know today I am in acute room. So, you really prepare yourself psychologically that I will really be busy but at least at the end of the day, I will evaluate myself and say, I did something for this baby.’ (IDI, Neonatal Nurse, Woman, Kenya)

Enhanced downward referrals to reduce non-complicated birth admissions during the pandemic were also seen on maternity wards. This also underscores the importance of strengthening primary and secondary health centres, which was highlighted by some respondents.

### Enhanced community engagement

An emerging theme was the desire for facilities providing SSNC to have stronger links with communities and increased community engagement. Stronger links would support follow up for babies who may have been discharged earlier due to the pandemic or due to ward closures. In Kenya, the closure of outpatient neonatal special clinics was mitigated by health workers initiative to contact the records department to follow up on extreme premature babies or pre-term babies who had been discharged.

Respondents reported more proactive messaging to communities and the importance of IPC measures, which was reported to have a positive effect on SSNC.

‘Before discharge, we sit down and we advise them… to restrict the numbers of people coming to the house, we also advise them to at least have a bucket of water and soap so that before anyone comes in or touches the baby should at least wash their hands, before taking care of the baby, but what we stress most is restrict people who come in because they may not know who is sick or not, who is COVID positive or not..’ (IDI, Clinician, Man, Malawi)

It was felt that the responsibility to engage with the community had increased since the pandemic, but that the power of community engagement had also increased. There were reports of messaging via televisions in hospitals, in Malawi linking with community gatekeepers who had recovered from COVID-19 to dispel myths and stigma in communities and in Tanzania, training and working with Community Health Workers (CHWs) to support community mobilisation. This was particularly critical given the context of scepticism of COVID-19 in Tanzania, which posed challenges in accepting IPC measures in hospital.

‘we need education to community, because you and I are both witnesses that there was a time in Tanzania used to believe that COVID, it is not for us, but it is here, so if the community was educated and equipped with knowledge….’ (IDI, Hospital Director, Tanzania)

### Enhanced maintenance of devices

Across all contexts, the pandemic created an enabling environment for enhanced focus on devices required for SSNC. There were examples of pre-emptive supply planning, such as proactively monitoring oxygen levels and ensuring they remain above a threshold or making allocation decisions on oxygen between wards or hospitals.

‘…we take capacity measurements every day so that we advise when it is below a certain point like now we don’t go below 20% of the capacity so that we give ourselves time between 20 and 10 to come and refill’. (IDI, Biomedical engineer, Woman, Kenya)

‘…because of the pandemic we have to do more of maintenance more frequently so that our equipment- number one they don’t break down and number two in case there is an issue we are able to get it faster, before it breaks down.’ (IDI, Neonatal Nurse, Woman, Kenya)

There was a renewed emphasis on planned preventive maintenance, in part attributed to the pandemic, but also due to NEST360 training and influence. This included a reduced turnaround time for maintenance of devices. Further strategies included the use of telemedicine to train on equipment use and increased communication between biomedical engineers and newborn units.

Despite the positive changes, critical limitations pertained to limited availability of locally available spare parts and a general lack of equipment to support SSNC, (monitors, incubators, infusion pumps, and autoclaving machines). Tragically one doctor in Nigeria narrated difficult ethical decisions taken to share oxygen among babies as there was limited supply of CPAP machines:

‘some of the equipment are limited, especially during the pandemic. At a point in time, we had like six or eight babies requiring ventilator support but in some of our wards we have three CPAP machines. So, we had to be withdrawing [babies] which is ethically not acceptable but we have to be weighing the benefits and the risks. So what we did was that babies who were relatively stable were withdrawn from the CPAP so that we could give access to the very sick babies.’ (IDI, Clinician, Man, Nigeria)

## Discussion

### Summary of main findings

Our study presents multi-country strategies employed across the health system during COVID-19 that served to protect small vulnerable newborns who were admitted to hospital. These were organised into two pathways (Fig. 3). Pathway one involved COVID-19 specific responses with a secondary benefitted for SSNC. This included increased collaboration and funding and improved IPC measures and support for oxygen systems among others. Pathway two involved health system strengthening strategies to mitigate negative effects of the pandemic, which may have also benefitted SSNC. This included changes to service delivery, HMIS, community engagement and human resource supportive strategies. Adaptations identified reveal opportunities to support routine newborn care if sustained post-pandemic. Our data also explicitly discuss critical gaps that require attention and investment to strengthen SSNC - such as a lack of mentorship among staff and the lack of locally available spare parts for device maintenance.

We found universal adaptations across the four contexts despite the different governance approaches to COVID-19 as reflected in the stringency indices (Fig. 1) [18]. In all contexts, new financing partnerships and governance arrangements were leveraged to support changes to devices, information systems and human resources, highlighting a systems approach to pandemic response. This occurred in a context of fear and anxiety for many within the health system. Building trust and connection with communities, and the provision of psychosocial support for health workers were other critical strategies to support a holistic pandemic response. We also identified novel differences between countries and facilities, providing unique learning. For example, health worker support structures demonstrated in Kenyan facilities or government support to establish oxygen plants in Tanzania could be adopted at scale.

### What do our findings mean for programmes, investment and future research?

**Pathway one:** COVID-19 specific responses that benefitted SSNC

Policy development and guidance: Our findings highlight the underlying inadequacy of neonatal-specific guidance in the face of COVID-19 and the need for integration of specific neonatal guidance into wider intervention strategies for health system shocks. COVID-19 led to initial disruptions to the mother-baby dyad that threatened family-centred and respectful care [6, 11, 21, 22]. Yet we found a lack of strategies identified to support family-centred or respectful care across all contexts. Respectful care is both a human rights and gender equity issue, and it is vital that policy responses to shocks uphold family-centred care [23]. Effective governance and communication channels between health-care providers and regional/national health authorities are crucial during disruptive events [21, 24] and were underlined by our respondents as a strength in the response. Critically though, these channels should provide communication opportunities from the bottom-up as well as top-down; health systems are inherently social institutions and enacting guidelines involves navigating the social tensions within health systems that may be heightened during a pandemic [24, 25]. Our data show the participation of key workers was limited. Inclusive policy development has been shown to support trust among healthcare workers, a critical component of effective health systems and essential in times of crisis, and ensures that guidelines align with workers’ values [24, 25]. Participatory approaches with health workers, families and communities to strengthen precautionary policies are required. This could support respectful care amid disease outbreaks [11], build trust and also bridge the gaps between community and facility that emerged in our findings.

New domestic and pooled funding streams and multi-sectoral collaboration identified in our study, allowed for system investments rather than funder specific investments, which served to strengthen the health system weaknesses that previously threatened SSNC [5]. Investments specifically in newborn care during COVID-19, however, were limited according to our data. For example, respondents in Malawi noted a lack of accepted funding proposals for neonatal care, despite the influx of COVID-19 funding and at the outset of the pandemic. This aligns with evidence that planning and budgeting for newborns is often dropped between maternal or child health [26]. Yet, investment in facility-based care for SSNC has been highlighted as a high-impact intervention with a major return on investment [27–29]. COVID-19 response catalysed synergistic opportunities for SSNC, such as the support for improved oxygen systems. Maintaining this support for piped oxygen as a standard of care across all facilities is critical for SSNC as our participants narrated the painful and heartbreaking decisions they make when there is not enough oxygen. Further research to explore power and decision making around financial priority setting at national and international scale would be valuable to understand how to support SSNC investments alongside broader strategies.

Strengthened IPC was reported to benefit SSNC and should be continuously evaluated to ensure it does not become an ad hoc outbreak response but is the standard of care. Improved IPC, however, needs to be balanced with the requirement to protect families’ decision-making powers as surveys have shown family-centred care practices were threatened by stringent IPC measures [6]. Multi-sectoral engagement to ensure enabling infrastructure (e.g. WASH) is critical, alongside investment in PPE, which requires consumption data for procurement and budgeting purposes.

**Pathway two**: Health system mitigation strategies and adaptations during the pandemic

Strengthening HMIS is a critical component of learning health systems to improve healthcare delivery [30] as well as for COVID-19 disease surveillance. Weak HMIS, and a lack of critical SSN indicators have also been identified as a bottleneck for improving SSNC [5]. Our study also highlighted a lack of data ownership and use within facilities sampled. For instance, the use of the Reproductive Maternal Newborn and Child Health scorecard was not highlighted as a data source to inform interventions at facility and national levels, despite having been adopted in our study countries, undermining accountability. Global evidence from disruptive events to health systems, demonstrated an interruption to wider HMIS during COVID-19 [16], however, our data suggest the focus on COVID-19 data collection may have supported a culture of informed decision making. This was particularly apparent in Tanzania, where respondents felt increased accountability due to government demand, oversight and training, highlighting the critical role of governance and education in changing culture. Our data, particularly from Nigeria, also highlight how investment in information technology systems, including mobile technologies, hardware and airtime, alongside wider systemic issues is key.

Human resource adaptations and supportive strategies to fill staffing gaps and boost morale were wide and varied across the facilities we spoke with. Some practices such as online training to support career and capacity development of staff, provision of financial incentives and task shifting were common across the countries. Task shifting has been recommended to support human resource for health shortages [5] and could be maintained with clear protocols in place. This, alongside some of the more novel practices employed may be a more sustainable way to incentivise staff than the provision of financial incentives, which elicited concerns and were not always distributed. Some more novel strategies included Malawi’s orientation programme. This was adopted in the COVID-19 response but could confer benefit to routine care, given the context of high staff turnover and rotation [31]. The provision of transport for health workers to address restrictions in movement in one Kenyan facility could also prove beneficial to staff and neonates as a lack of transport has been shown to be a barrier to healthcare delivery for neonates during travel restrictions [32]. This was provided alongside insurance and mental health support, which are critical areas of consideration. The stigmatization of COVID-19 and the mental health impact among health workers have manifested as experiences of trauma and confusion [33]. Health systems have a duty of care to health workers [34]; holistic support for health workers may include counselling, mentoring, and supportive supervision. This should be extended beyond health systems shocks to ensure all health workers are adequately supported as a routine component of their jobs as caregivers and to reduce burn-out, a global issue among health workers but particularly pertinent in crises. Implementation and participatory research is needed to identify strategies to support health worker wellbeing and mental health and heed the call of increased mentorship from our respondents.

Service delivery changes and innovations occurred across the health system in the pandemic and we report on changes within SSNC wards, including separation by dependency status, which was reported to reduce infection spread and improve staff morale. Respondents spoke of using telemedicine to support continuity of neonatal care during the COVID-19 pandemic, for training and for device maintenance. Qualitative data on telemedicine use during previous pandemics has largely only been shown in high income country settings to date [35], though its use during obstetric and maternal care during lockdown settings has been evidenced in LMIC to alleviate healthworker fear [15, 36, 37]. There is little data to support best practices for telemedicine that ensure a standard of care is maintained, especially for small and sick neonates who are particularly vulnerable [37]. Key considerations emerging from our data relate to the need for staff training on software use and the requirement for air-time to ensure this cost is not borne by staff. This was also implied to have equity considerations for human resources where younger staff may have to choose between paying for PPE to protect themselves, or airtime to protect their patients. These concerns have also been documented in global survey data [38]. The sustainability, feasibility and mechanism of provision of airtime direct to staff or facilities requires exploration to ensure inequities and unintended consequences are not created.

Increased community engagement and referrals was another call from our respondents and a fundamental strategy for infectious disease control [39]. Working together with CHWs and community leaders was a strategy used by facilities to dispel myths and misconceptions to support continuity of care in a context of fear. These linkages with communities, and innovations in communication platforms, e.g., television, social media, could be leveraged beyond health system shocks for ongoing patient education on routine newborn care practices. Creating stronger, institutionalised referral structures is also important to support follow up and avoid the burden of follow-up in health system shocks being put on individual healthcare workers, as we found in Kenya.

Enhanced device maintenance: A renewed emphasis on planned preventive maintenance since the pandemic through structured means of reporting and accountability mechanisms was a positive emerging from our data, though may have also been influenced by ongoing work in NEST360 on this theme. Nonetheless, the availability of spare parts and appropriate consumables were ubiquitous challenges. Addressing them requires effective and continuous dialogue between biomedical technicians, procurement staff and device manufacturer/distributors. Further, empowering local production of medical supplies will progress to a more sustainable model of device maintenance and reduce life-threatening delays in procuring spare parts.

### Strengths and limitations of our approach

Our findings are largely consistent with broader global literature on disruptions and mitigation strategies for SSNC following COVID-19 [6, 9, 16, 22, 32], but extend this to provide qualitative evidence across four African settings. This is essential to inform strategies for addressing future health system shocks (including from pandemics) and share lessons learnt from across multiple country contexts. This research collected a large dataset from across four countries with NEST360. We co-developed discussion guides with the wider NEST360 team, including clinicians, policymakers, biomedical technicians and experts in SSNC to ensure guides were grounded in contextual realities. The common guides and co-analysis process enabled a shared coding framework to compare across the four countries highlighting similarities and differences, a key strength of this research. A key limitation is the fact data were collected from facilities in which NEST360 was working to strengthen device maintenance and information systems, which are two of the areas reported in this study, though themes such as IPC and policy development likely had resonance across other facilities. Respondents working at the national level would also have oversight beyond NEST360 facilities. Further, the position of NEST360 being a trusted partner with funds had the potential to bias the results. Weekly reflexivity sessions held among the team meant that our positionality as a part of NEST360 was explored and included in our analysis process. Further, the team felt that the strong rapport they built with respondents allowed for open and honest reflection on how the pandemic was handled on the ground. Finally, data in Tanzania were collected later, which may have resulted in missed opportunities for the Tanzanian emerging findings to input into the other countries' data collection. However, the lead researcher in Tanzania (JB) was involved in all stages research prior, including the co-development of the coding framework and was able contribute observations from Tanzania.

## Conclusion

Building stronger and more resilient SSNC that supports a family-centred approach requires leveraging investment in pandemic preparedness to support health system strengthening. COVID-19 shone a spotlight on health system weaknesses and required rapid change, adaptation and innovation to maintain services. Harnessing and learning from this will help build resilience in SSNC. This requires deliberate efforts to protect gains made, address gaps and secure additional strategic investments to support sustainability. Investment cases for SSNC that build on identified mitigation strategies and embed these within existing systems will ensure vulnerable newborns are able to survive and thrive even when facing future health system shocks.

## Supplementary Information

**Additional file 1.**

## Data Availability

Data sharing and transfer agreements were jointly developed and signed by all collaborating partners. The anonymised transcripts can be accessed subject to approval by collaborating parties.

## Abbreviations

COVID-19: Coronavirus 19 (Severe acute respiratory syndrome coronavirus 2)
CHWs: Community Health Workers
ENAP: Every Newborn Action Plan
FGD: Focus Group Discussion
HMIS: Health Management Information System
IDI: In-depth Interview
IPC: Infection Prevention Control
KMC: Kangaroo Mother Care
LMIC: Low-and middle-income countries
LSHTM: London School of Hygiene & Tropical Medicine
NEST360: Newborn Essential Solutions and Technologies
OxCGRT: Oxford COVID-19 Government Response Tracker
PPE: Personal Protective equipment
SDGs: Sustainable Development Goals
SSN: Small and sick newborns
SSNC: Small and sick newborn care
WHO: World Health Organisation

## Declarations

Ethics and consent to participate
