# Supplementary material for: Protecting small and sick newborn care in the COVID-19 pandemic: multi-stakeholder qualitative data from four African countries with NEST360
Source: BMC Pediatr. 2023 Nov 16;23(Suppl 2):572. doi: 10.1186/s12887-023-04358-7 (PMC10655439; doi:10.1186/s12887-023-04358-7)
Supplement: Supplementary file 1 — Additional file 1. [file 12887_2023_4358_MOESM1_ESM.docx]

Supplementary files NEST360 COVID-19 qual paper

Supplementary file 1:

Daily cases and deaths across the four countries from the period March 2020 – Sept 2021. These data are taken from WHO COVID-19 Dashboard but are noted to be unreliable.


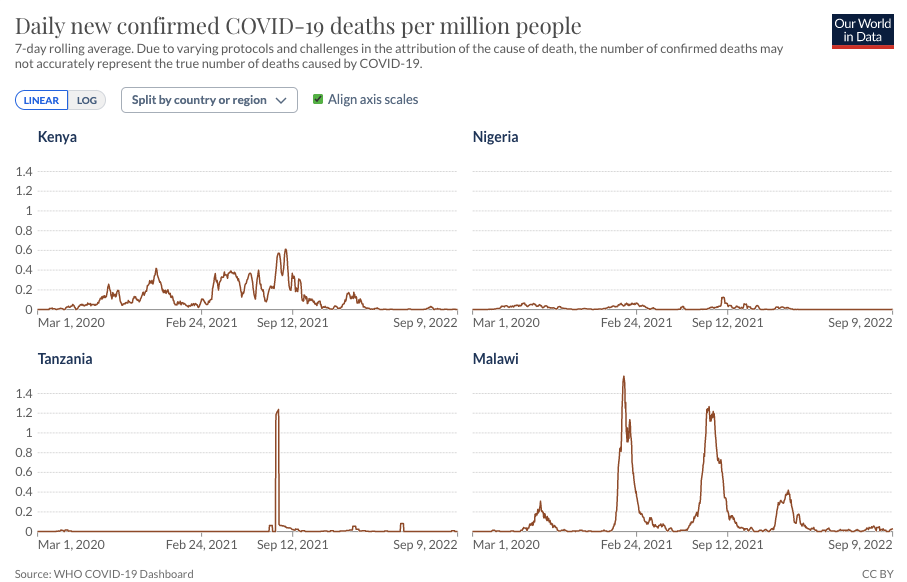


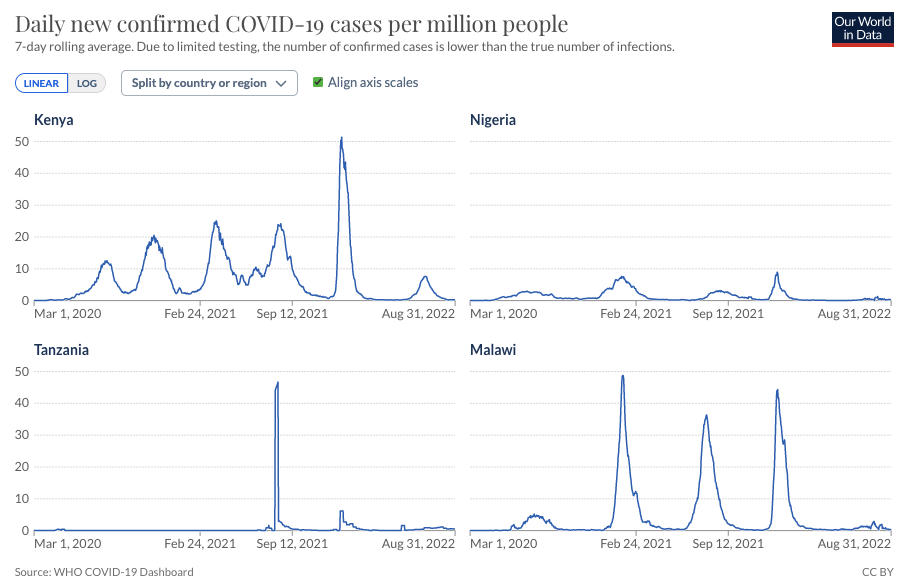


Supplementary file 2:

**NEST360 COVID-19 Mitigation Study Form – In Depth Interview guide for policy makers/ mid level management/ stakeholders and Nest 360 staff**

| **Interviewee Code** |  |
| --- | --- |
| **Institution** |  |
| **Place and date** |  |
| **Position** |  |
| **Age band** |  |
| **Gender** |  |
| **Period worked on NBU** |  |

**Set up**

**Please take informed consent & find a private, comfortable location. Or if online, somewhere private and with stable internet connection to allow for video on.**

Introduction: Hello, my name is………….. and I am here on behalf of the Newborn Essential Solutions and Technologies (NEST) program. The programme’s goal is to ensure every hospital in Africa can deliver life-saving care for small and sick newborns. As you know the COVID pandemic has disrupted SSNC and health systems globally. The purpose of today’s interview is to understand about protection of SSNC services under covid. You may or may not work on newborn care but we would like to learn about what ideas and innovations are taking place in your context to support protection of these services.

As explained in the study information sheet. The interview is voluntary and you are free to withdraw your right to participate at any time. If any questions make you uncomfortable please let me know and we can skip them.

**Policy level suggested respondents: National MNH coordinator, National health insurance, National quality**

1. **Introduction / Warm up**
   1. Can you describe your role?
   2. Has this changed compared to the period before the pandemic? How?
2. **Policy Development**
   1. How were new covid policies/ guidelines on SSNC developed?
      1. *What changes were made? What is your opinion of these changes?*
      2. *Who were the actors involved?*
      3. *What was the consultation process with hospital staff/ communities (if any?)*
      4. *If you could go back and change anything in terms of policy development, would you? Why?*
   2. Were there any unintended consequences of covid policies?
      1. *Please describe…*
      2. *How might you mitigate against that?*
   3. Do you think any of the new policies or guidelines should remain in place long-term, why?
      1. *Do you think they could be improved?*
   4. What was the role of of other national level organisations? (professional bodies etc)
3. ***Service delivery***

How did national level managers support organizations of care (PHCs, SHC & THC facilities) and commodities for newborn care?

*Probe: advocacy, guideline development, fundraising*

*Can you describe any changes in attitudes of facility level staff since the pandemic?*

1. **Data and Information Use**
   1. What data was used to support policy development?
      1. *How are the data on SSNC disaggregated?*
      2. *In what ways were factors relating to inequity considered? e.g. age, Socio-Economic Status, education, gender*
   2. What changes in data/reporting did you ask for/ were asked for from facilities/mid-level management teams?
      1. *Demand/ speed of reporting?*
      2. *How were these data shared? Were they integrated into the national HMIS?*
      3. *Electronic means?*
      4. *Did you use covid data in any way?*
   3. How might COVID data be represented differently to highlight service interruptions within SSNC?
   4. Can you comment on changes in completeness or quality of data?
   5. What information would you like to have about the (newborn) services you provide that you don’t have now? How would this help the health system/facilities provide better care?
2. **Investment and macro-strategies (skip for mid-level mgmt.)**
   1. Which actors had the greatest impact on mobilising resources or implementing high impact interventions for covid?
      1. *How about for HSS more generally?*
      2. *How do you work with them effectively?*
   2. What needs for additional resources were identified in the course of the pandemic?
      1. *Have these been met or not? By whom?*
   3. Have you seen the introduction of new funding (private sector, industry) or interesting resource pooling? Describe.
   4. How has financial protection affected care-seeking in the covid era? How do you think it would be best to mitigate these effects?
      1. *Is there anything specific that has been learned that could be sustained to influence SSNC care-seeking, service delivery?*
3. **Partnerships and Collaborations**
   1. How have collaborations across units/teams in facilities been effective to strengthen newborn care delivery in the covid era?
      1. *Probe for: with ICU, with pediatrics, with biomed*
   2. How have you collaborated across sectors to strengthen newborn care delivery in the covid era?
      1. *Probe for: with education, security, WASH, ‘unusual suspects’*
   3. Have there been any interesting collaborations with industry/private sector that might provide a model for future work?
4. **Key concluding questions:**
   1. What, in your opinion, are the most important practices in maintaining quality newborn care that have emerged during pandemic?
      1. *Probe – are these practices reflected in policy?*
      2. *Was there a particular area you felt you had to prioritise?*
   2. How do you want things to continue to change for SSNC?
   3. Is there anything you’ve heard of, or thought about, that you would have liked to have done/implemented if resources were not a constraint?

**For NEST 360 Staff only:**

1. How was the availability of consumables & supplies for SSNC affected by COVID?
   1. Where these diverted?
   2. Challenges in distribution of devices?
   3. What was done to overcome these challenges/changes?
2. What role did Nest play in identifying solutions and protecting SSNC?
   1. How were solutions identified?
   2. Who brokered/ catalysed these solutions?

Finally, thank you for your time, is there anything else you would like to share with me today?

**NEST360 COVID-19 Mitigation Study Form – In Depth Interview guide for funders**

| **Interviewee Code** |  |
| --- | --- |
| **Institution** |  |
| **Place and date** |  |
| **Position** |  |
| **Age** |  |
| **Gender** |  |
| **Period worked on NBU** |  |

**Please take informed consent & find a private, comfortable location.**

Introduction: Hello, my name is………….. and I am here on behalf of the Newborn Essential Solutions and Technologies (NEST) program. The programme’s goal is to ensure every hospital in Africa can deliver life-saving care for small and sick newborns. As you know the COVID pandemic has disrupted SSNC and health systems globally. The purpose of today’s interview is to understand about protection of SSNC services under covid. You may or may not work on newborn care but we would like to learn about what ideas and innovations are taking place in your context to support protection of these services.

As explained in the study information sheet. The interview is voluntary and you are free to withdraw your right to participate at any time. If any questions make you uncomfortable please let me know and we can skip them.

1. **Warm up**
   1. Can you describe your role?
   2. Has this changed compared to the period before the pandemic? How?

**2. Investment and macro-strategies**

- 1. Which actors had the greatest impact on mobilising resources or implementing high impact interventions for covid?
     1. *How about for HSS more generally?*
     2. *How do you work with them effectively?*
  2. What needs for additional resources were identified in the course of the pandemic?
     1. *Have these been met or not? By whom?*
  3. Have you seen the introduction of new funding (private sector, industry) or interesting resource pooling? Describe.
  4. How has financial protection affected care-seeking in the covid era? How do you think it would be best to mitigate these effects?
     1. *Is there anything specific that has been learned that could be sustained to influence SSNC care-seeking, service delivery?*
  5. How are you thinking about quality of SSNC when making funding decisions?
     1. *In terms of the software of health systems (staff training, mentorship, wellbeing) & hardware*
     2. *Did you change your funding system to support protection of services? E.g. emergency provisions (for newborns and otherwise)*

1. **Partnerships and Collaborations**
   1. Did you fund differently, or collaborate with other funders during the pandemic?
      1. *What was the impact of this?*
   2. How have collaborations across units/teams in facilities been effective to strengthen newborn care delivery in the covid era?
      1. *Probe for: with ICU, with pediatrics, with biomed*
   3. How have you collaborated across sectors to strengthen newborn care delivery in the covid era?
      1. *Probe for: with education, security, WASH, unusual suspects*
   4. Have there been any interesting collaborations with industry/private sector that might provide a model for future work?
2. **Key concluding questions:**
   1. What, in your opinion, are the most important practices in maintaining quality newborn care that have emerged during pandemic?
      1. *Probe – are these practices reflected in policy?*
      2. *Was there a particular area you felt you had to prioritise?*
   2. How do you want things to continue to change for SSNC?
   3. Is there anything you’ve heard of, or thought about, that you would have liked to have done/implemented if resources were not a constraint?

Thank you for your time, is there anything else you would like to share with me?

**NEST360 COVID-19 Mitigation Study Form – In Depth Interview guide for facility staff**

| **Interviewee Code** |  |
| --- | --- |
| **Institution** |  |
| **Place and date** |  |
| **Position** |  |
| **Age** |  |
| **Gender** |  |
| **Period worked on NBU / in hospital** |  |

**Please take informed consent & find a private, comfortable location.**

Introduction: Hello, my name is………….. and I am here on behalf of the Newborn Essential Solutions and Technologies (NEST) program. The programme’s goal is to ensure every hospital in Africa can deliver life-saving care for small and sick newborns. As you know the COVID pandemic has disrupted SSNC and health systems globally. The purpose of today’s interview is to understand about protection of SSNC services under covid. You may or may not work on newborn care but we would like to learn about what ideas and innovations are taking place in your context to support protection of these services.

As explained in the study information sheet. The interview is voluntary and you are free to withdraw your right to participate at any time. If any questions make you uncomfortable please let me know and we can skip them.

Key:

Clinicians only

Biomed only

HMIS people only

- - - 1. **Warm up**
  1. Can you describe your role?
  2. How has this changed compared to the period before the pandemic?
  3. How has the way people work changed?
     1. *on the ward, within the hospital?*
     2. *What about relationships with mothers/families?*

1. **Organization of Care and Systems**
   1. How do you think the mid-level managers (CHMT/DHMT) and senior facility staff handled the delivery of small and sick newborn care in the COVID-19 pandemic? Can you give an example?
      1. *What did they do well? Did they support smooth delivery?*
      2. *What did not go so well?*
      3. *What were some of the challenges/ unintended consequences faced?*
      4. *What were the successes?*
      5. *new policy roll out- how were any myths & misconceptions addressed?*
      6. *Where these actions supported/ reflected in policy?*
   2. Has the hospital had to reorganise how it operated eg. changing the use of different wards or visiting hours or restrictions? Can you tell me a bit about how they have tried to do this?
   3. How did covid policies/guidelines get operationalised?
      1. *Do you think it has been successful - Why / Why not?*
      2. *What other changes would you have liked to see?*
      3. *Did you have any input into decision making/policy development? Can you describe this…*
   4. Have there been changes in the equipment and ways of working?
      1. Probe:*e.g to oxygen supplies or KMC; change in visiting hours for care-giver, ward layouts, IPCd*
      2. *What was the impact of these changes to patients?*
      3. *Can you describe any changes to patient messaging?*

*Probe: Were stigma, myths addressed?*

- 1. Can you describe any new ways of working to support SSNC at the facility level?
  2. Has any new telemedicine been introduced?
     1. *What are the pros and cons here?*
     2. *How do you identify broken equipment virtually/how is it resolved?*
  3. Have there been any new ways of working to support continuing care at the community level?
     1. *Probe: community follow ups, transport - pros and cons?*
  4. What was your involvement in changes to service delivery OR HMIS systems OR Maintenance of technologies since the pandemic?
     1. *Probe: Did you get training? were there new things to learn? is the equipment easy to use and do you feel confident with this?*
     2. *What were the reactions of people to these changes?*

1. **Capacity**
   1. Can you describe how COVID impacted on health workers?
   2. What system level changes to standards and recruitment occurred?
      1. *Staff re-deployment? Staff absence? Incentives?*
      2. *Any mitigation strategies to address changes in staffing?*
   3. Can you describe any supervision and mentorship approaches across levels to address health worker burnout etc.?
   4. Any (other) wellbeing approaches to discuss?
   5. How have team structures been re-thought?
      1. *Was there new task sharing? Pros/cons of this*
      2. *How did you foster new collaborations?*
   6. Can you tell me about any staff training that occurred?
      1. *Have there been some/more interdisciplinary trainings to address new needs in oxygen systems etc.? (eg clinical + biomedical engineering)*
      2. *New approaches here – remote, digital?*
      3. *Content*
      4. *Who was responsible? Who was involved?*
      5. *Are there further capacity needs related to newborn care illuminated by covid that are unmet? Describe.*
      6. *Was training in response to covid or existing weaknesses illuminated by covid?*
2. **New Hardware, Systems and Resources**
   1. What new hardware and systems have been introduced as a result of covid?
      1. *Will this be needed in the current position long-term (for how long)?*
      2. *Where will it be used beyond peak of covid?*
      3. *How could the newborn ward benefit from this?*
   2. Tell us more about Oxygen systems specifically
   3. Tell us more about handwashing devices/systems for IPC?
   4. What other resources have been introduced?
      1. *By whom?*
      2. *Is there a plan for this to be sustained? Should it be?*
   5. *For maintenance of devices, what is the typical turn-around time for problems to be identified and corrected?*
   6. How has the maintenance of devices occurred?
      1. *Probe: Has it changed? Remote innovations? Challenges?*
      2. *Probe: same for Preventative vs. corrective maintenance*
   7. How have you provided user training to clinical staff during the pandemic?
      1. *Probe: any remote innovations/ changes here? Challenges?*
3. **Data and Information Use**
   1. What changes in data/reporting were required?
      1. *Demand/ speed of reporting?*
      2. *How were these data shared? Electronic means? Innovations here?*
      3. *How are the data disaggregated? In what ways were factors relating to inequity considered? e.g. age, SES, education*
   2. Can you comment on changes in completeness or quality of data?
   3. What information would you like to have about the services you provide that you don’t have now? Why?
4. **Conclusion**
   1. What, in your opinion, are the most important practices in maintaining quality newborn care that have emerged during pandemic?
   2. How do you want things to continue to change for SSNC?

Thank you so much for your time, is there anything else you would like to share with me?

**NEST360 COVID-19 Mitigation Study Form – In Depth Interview guide for device distributors/procurement system**

| **Interviewee Code** |  |
| --- | --- |
| **Institution** |  |
| **Place and date** |  |
| **Position** |  |
| **Age** |  |
| **Gender** |  |
| **Period worked on NBU / in hospital** |  |

**Please take informed consent & find a private, comfortable location.**

Introduction: Hello, my name is………….. and I am here on behalf of the Newborn Essential Solutions and Technologies (NEST) program. The programme’s goal is to ensure every hospital in Africa can deliver life-saving care for small and sick newborns. As you know the COVID pandemic has disrupted SSNC and health systems globally. The purpose of today’s interview is to understand about protection of SSNC services under covid. You may or may not work on newborn care but we would like to learn about what ideas and innovations are taking place in your context to support protection of these services.

As explained in the study information sheet. The interview is voluntary and you are free to withdraw your right to participate at any time. If any questions make you uncomfortable please let me know and we can skip them.

1. **Warm up**
   1. Can you describe your role?
   2. Has this changed compared to the period before the pandemic? How?
2. **New Hardware, Systems and Resources**

- How was the availability of consumables & supplies for SSNC affected by COVID?
  - Where these diverted?
  - Challenges in distribution of devices?
  - What was done to overcome these challenges/changes?
- What was your (/organisations) involvement in changes to procurement/distrbution since the pandemic?
- What new hardware and systems have been introduced as a result of covid?
  - Can you describe how these changes arose?
  - Who was involved in catalysing these changes?
  - Will this be needed in the current position long-term (for how long)?
  - Where will it be used beyond peak of covid?
  - How could the newborn ward benefit from this?
  - Tell us more about Oxygen systems specifically
  - Tell us more about handwashing devices/systems for IPC?
- What other resources have been introduced?
  - - *By whom?*
    - *Is there a plan for this to be sustained? Should it be?*
- For maintenance of devices, what is the typical turn-around time for problems to be identified and corrected?
  - How has the maintenance of devices occurred?
    - *Probe: Has it changed? Remote innovations? Challenges?*
    - *Probe: same for Preventative vs. corrective maintenance*

1. **Organisation of care systems**

- How do you think the mid-level managers (CHMT/DHMT) and senior facility staff handled the delivery of small and sick newborn care in the pandemic? Can you give an example?
  - *What went well?*
  - *What did not go so well?*
  - *What were some of the challenges/ unintended consequences faced?*
  - *What were the successes?*
  - *new policy roll out - how were any myths & misconceptions addressed?*
  - *Where these actions supported/ reflected in policy?*
- Has the hospital had to reorganise how it operated? Can you tell me a bit about how they have tried to do this?
- Have there been changes in the equipment and ways of working?
  - 1. Probe:*e.g to oxygen supplies or KMC; change in visiting hours for care-giver, ward layouts, IPCd*
    2. *What was the impact of these changes?*

**Conclusion**

- 1. What, in your opinion, are the most important practices in maintaining quality newborn care that have emerged during pandemic?
  2. How do you want things to continue to change for SSNC?

Thank you for your time is there anything else you would like to share with me?

**NEST360 COVID-19 Mitigation Study Form – Focus group discussion guide for hospital staff at facility level**

| **Interview Code** |  | | | | | | | |
| --- | --- | --- | --- | --- | --- | --- | --- | --- |
| **Hospital** |  | | | | | | | |
| **Place and date** |  | | | | | | | |
| **Position** |  |  |  |  |  |  |  |  |
| **Age** |  |  |  |  |  |  |  |  |
| **Gender** |  |  |  |  |  |  |  |  |
| **Period worked on NBU / in hospital** |  |  |  |  |  |  |  |  |

**Instuctions for use**

After introducing yourself, explaining the purpose of the interview, seeking consent and finding a location that will make the interviewees comfortable

Facility/Ward level (Target: Doctors, nurses, bio-medical engineers, hospital management (HMT))

Introduction

Hello, my name is………….. and I am here on behalf of the Newborn Essential Solutions and Technologies (NEST) program. The programme’s goal is to ensure every hospital in Africa can deliver life-saving care for small and sick newborns. As you know the COVID pandemic has disrupted SSNC and health systems globally. The purpose of today’s interview is to understand about protection of SSNC services under covid. You may or may not work on newborn care but we would like to learn about what ideas and innovations are taking place in your context to support protection of these services.

As explained in the study information sheet. The interview is voluntary and you are free to withdraw your right to participate at any time. If any questions make you uncomfortable please let me know and we can skip them. Please also note that anything shared in the group should not be shared outside of this space – so that we are all free to speak openly.

Clinicians only

Biomed only

HMIS people only

1. **Introduction / Warm up**
   1. What areas have you seen the biggest changes in facility-based SSNC during covid?
      1. probe for: visitation, mother-baby separation, organization of care, access to services, time to discharge, care seeking

b. Do communities still come to the hospital as much as they did?

1. What opportunities have emerged during covid that have resulted in improvement in care? Can these be sustained? What would that take?
2. What existing weakenesses were highlighted by covid and how did you respond?
3. **Policy interventions**
   1. Thinking back to the beginning of covid, what were some of the covid policy/guideline changes that occurred?
   2. Who/which levels/departments/organizations are leading the policy response to covid?
   3. Were policies implemented successfully? How could they be improved?
   4. Can you tell me about any unintended consequences from new policies on SSNC?
      1. *Probe: Community level e.g. access to care, health seeking, respectful care, accountability, financing*
      2. *Facility level?*
      3. *What were the feedback mechanisms in place for assessing new policies on SSNC?*
   5. Have there been any benefits to these policies? Do you think any of these policies should be sustained? Why?
      1. *Probe: influence on SSNC care-seeking, service delivery, financing*
4. **Organization of Care and Systems**
   1. How did senior staff respond to changes in delivery of small and sick newborn care?
      1. What did they do well? What did not go so well?
      2. Probe: new policy roll out/ addressing myths & misconceptions
   2. Have there been changes in the equipment and ways of working?
      1. Probe:*e.g to oxygen supplies or KMC or CPAP; change in visiting hours for care-giver, ward layouts, IPCd*
      2. *How was patient engagement/messaging and addressing stigma,myths incorporated?*
   3. Has the hospital had to reorganise how it operated eg. changing the use of different wards or visiting hours or restrictions? Can you tell me a bit about how they have tried to do this?
      1. *Probe: changes to physical spaces/wards etc.*
5. **Capacity**
   1. What were some of the solutions to get around changes in health workers?
      1. *Staff re-deployment? Recruitment?*
      2. *How were teams restructured?Was there new task sharing?*
      3. *How did you collaborate with others*
   2. How did engagement with management happen around this?
      1. Were there opportunities to input into management decisions? What was this process?
   3. Were there any new approaches to mitigate against changes to staffing? Please explain…
      1. *Were these on the ground changes, or reflected in policy?*
      2. *Who were the decision makers*
      3. *Any peer support mechanisms?*
   4. Can you tell me about any training undertaken and whether it was in response to covid or existing weaknesses/opportunities illuminated by covid?
      1. *Innovations/ new approaches here – remote,digital?*
      2. *Content*
   5. Can you describe any general wellbeing support mechanisms implemented?
      1. *Pros/cons*
6. **New Hardware, Systems and Resources**
   1. What hardware/equipment was introduced into the facility? Where and why did this happen?
      1. *Will this be needed here long-term?*
      2. *Where will it be used beyond peak of covid?*
      3. *How could the newborn ward benefit from this?*
   2. Tell us more about Oxygen systems specifically
   3. Tell us more about handwashing devices/systems for IPC?
   4. What other resources have been introduced?
      1. *By whom?*
      2. *Is there a plan for this to be sustained? Should it be?*
   5. Can you decsirbe how maintenance of equipment occurred – any innovations here?
      1. How were faulty devices identified & fixed
      2. Timescale of maintenance
   6. How was training on devices conducted?
7. **Data and Information Use**
   1. How did the demand for and use of data change from normal reporting?
      1. *In speed of reporting*
      2. *Electronic means?*
      3. *Did you use covid data in any way?*
   2. Can you comment on completeness or quality of data?
   3. What information would you like to have about the services you provide that you don’t have now? Why?
8. **Investment and macro-interventions (if time)**
   1. Have you seen the introduction of new funding (private sector, industry) or interesting partnerships?
   2. Anything that could be sustained to influence care-seeking, service delivery
9. **Partnerships and Collaborations (if time)**
   1. Can you describe the role of other departments in protecting and mitigating SSNC disruptions?
      1. What they did well/ what they didn’t do well?
      2. Did the Maternity/labour ward and MCH departments implement any COVID-19 related interventions and did this affect care for newborns?

**Conclusion**

- 1. What else have you as a team learned from covid?
  2. In your opinion, What other interventions should be put in place to ensure quality newborn care including at the community?
  3. Is there anything you’ve heard of, or thought about, that you would have liked to have done/implemented if resources were not a constraint?
  4. Finally, how do you want things to continue to change?

Thank you for your time, is there anything else you would like to share with me?

Do participant checking, reassure about confidentiality

**NEST360 COVID-19 Mitigation Study Form – Focus group discussion guide for Funders/Stakeholders/ Nest 360 team**

| **Interview Code** |  | | | | | | | |
| --- | --- | --- | --- | --- | --- | --- | --- | --- |
| **Hospital** |  | | | | | | | |
| **Place and date** |  | | | | | | | |
| **Position** |  |  |  |  |  |  |  |  |
| **Age** |  |  |  |  |  |  |  |  |
| **Gender** |  |  |  |  |  |  |  |  |
| **Period worked on NBU** |  |  |  |  |  |  |  |  |

**Instuctions for use**

After introducing yourself, explaining the purpose of the interview, seeking consent and finding a location that will make the interviewees comfortable

Introduction

Hello, my name is………….. and I am here on behalf of the Newborn Essential Solutions and Technologies (NEST) program. The programme’s goal is to ensure every hospital in Africa can deliver life-saving care for small and sick newborns. As you know the COVID pandemic has disrupted SSNC and health systems globally. The purpose of today’s interview is to understand about protection of SSNC services under covid. You may or may not work on newborn care but we would like to learn about what ideas and innovations are taking place in your context to support protection of these services.

As explained in the study information sheet. The interview is voluntary and you are free to withdraw your right to participate at any time. If any questions make you uncomfortable please let me know and we can skip them. Please also note that anything shared in the group should not be shared outside of this space – so that we are all free to speak openly.

1. **Introduction / Warm up**
   1. What areas have you seen the biggest changes in facility-based SSNC during covid?
      1. probe for: visitation, mother-baby separation, organization of care, access to services, time to discharge, care seeking
   2. What opportunities have emerged during covid that have resulted in improvement in care? Can these be sustained? What would that take?
   3. What existing weakenesses were highlighted by covid and how did you respond?
2. **Policy interventions**
   1. Thinking back to the beginning of covid, what were some of the covid policy/guideline changes that occurred?
   2. Who/which levels/departments/organizations are leading the policy response to covid?
      1. Probe – What was the process of policy development? Who was involved?
      2. Have any interesting partnerships been generated?
      3. Were policies implemented successfully? How could they be improved?
   3. Can you tell me about any unintended consequences from new policies on SSNC?
      1. *Probe: Community level e.g. access to care, health seeking, respectful care, accountability, financing*
      2. *Facility level?*
      3. *What were the feedback mechanisms in place for assessing new policies on SSNC?*
   4. Have there been any benefits to these policies? Do you think any of these policies should be sustained? Why?
      1. *Probe: influence on SSNC care-seeking, service delivery, financing*
   5. What data was used to aid decision making?
      1. Probe: quality, timely?
      2. How was data disaggregated?
3. **Organization of Care and Systems (skip for funders)**
   1. How did hospitals respond to changes in delivery of small and sick newborn care?
      1. What did they do well?
      2. What did not go so well?
      3. Probe: new policy roll out/ addressing myths & misconceptions
4. **Capacity (skip for funders)**
   1. Were there any innovations to mitigate against changes to staffing? Please explain…
      1. *Were these on the ground changes, or reflected in policy?*
      2. *Who were the decision makers*
      3. *Any peer support mechanisms?*
   2. Can you tell me about any training undertaken?
      1. Probe: was this in response to covid or existing weaknesses/opportunities illuminated by covid?
      2. *New approaches here – remote,digital?*
      3. *Content*
      4. *Who was responsible? Who was involved?*
   3. Do you know of any general wellbeing support mechanisms implemented?
5. **New Hardware, Systems and Resources**

How was the availability of consumables & supplies for SSNC affected by COVID?

- 1. Where these diverted?
  2. Challenges in distribution of devices?
  3. What was done to overcome these challenges/changes?

What hardware/equipment was introduced into the facility? Where and why did this happen?

- - 1. *Who was responsible?*
    2. *Will this be needed here long-term?*
    3. *How was the hardware procured? Where will it be used beyond peak of covid?*
    4. *How could the newborn ward benefit from this?*
  1. Tell us more about Oxygen systems specifically
  2. Tell us more about handwashing devices/systems for IPC?
  3. What other resources have been introduced?
     1. *By whom?*
     2. *Is there a plan for this to be sustained? Should it be?*

1. **Data and Information Use**
   1. How did the demand for and use of data change from normal reporting?
      1. *In speed of reporting*
      2. *Electronic means?*
      3. *Did you use covid data in any way?*
   2. Can you comment on the quality of data?
   3. How might data reporting be improved to better capture interruption of services from COVID and other health system shocks?
2. **Investment and macro-interventions**
   1. Which actors had the greatest impact on mobilising resources or implementing high impact interventions for covid? For HSS more generally?What are the needs for additional resources (met or not? By whom?)
   2. Have you seen the introduction of new funding (private sector, industry) or interesting partnerships?
   3. Anything that could be sustained to influence care-seeking, service delivery
      1. Having to pay for care even if financial protection
3. **Partnerships and Collaborations**
   1. Can you describe the role of other departments in protecting and mitigating SSNC disruptions?
      1. What they did well/ what they didn’t do well?
      2. Did the Maternity/labour ward and MCH departments implement any COVID-19 related interventions and did this affect care for newborns?

**For NEST 360 Staff only:**

1. What role did Nest play in identifying solutions and protecting SSNC?
   1. How were solutions identified?
   2. Who brokered/ catalysed these solutions?

b. Key **Concluding questions**

- 1. What other interventions should be put in place to ensure quality newborn care including at the community?
  2. What else have you learned from covid?
  3. How do you want things to continue to change?
  4. Is there anything you’ve heard of, or thought about, that you would have liked to have done/implemented if resources were not a constraint?

**Thank you so much for your time, is there anything else you’d like to share with me?**

Do participant checking, reassure about confidentiality
